# Safety and Efficacy of Ultrasound-Accelerated Endovascular Lysis in Postoperative Patients with Intermediate–High-Risk Pulmonary Embolism: A Retrospective Two-Center Study

**DOI:** 10.3390/jcm15072600

**Published:** 2026-03-29

**Authors:** Abdelrahman Elhakim, Martin Knauth, Mohamed Elhakim, Osama Bisht, Jan-Erik Guelker, Hani Al-Terki

**Affiliations:** 1 Schoen Hospital, 23730 Neustadt in Holstein, Germany; mknauth@schoen-klinik.de; 2 Intensive Care Medicine Department, The Royal Prince Alfred Hospital, 50 Missenden Rd, Camperdown, Sydney, NSW 2050, Australia; myelhakim@hotmail.com; 3 Coswig Heart Center, 06869 Coswig, Germany; obisht@gmail.com; 4 Cardiology Department, Petrus Hospital, 42283 Wuppertal, Germany; jan-erik.guelker@cellitinnen.de (J.-E.G.); hani1990@gmx.de (H.A.-T.); 5 Cardiology Department, University Witten/Herdecke, 58455 Witten, Germany

**Keywords:** pulmonary embolism, postoperative, bleeding, complications, catheter-based therapy

## Abstract

**Background:** Postoperative patients are at risk of pulmonary embolism. They typically exhibit multiple contributing factors such as comorbidities, immobility, blood loss, increased hematocrit, dehydration, long hospital stays, and a higher bleeding risk. PE management in this vulnerable group is challenging. Although current guidelines provide differing recommendations, many clinical questions remain unanswered. Decisions regarding periprocedural anticoagulation management must balance the thromboembolic and procedural higher bleeding risks. In addition, a recent major surgery is an absolute contraindication to systemic thrombolysis. Small doses of local lytics or a mechanical percutaneous embolectomy in the era of catheter-based therapy may be a safer option. However, the safety and efficacy of CDT have not been evaluated in this particular PE-vulnerable population. **Methods:** We performed a retrospective study of 35 postoperative patients with intermediate–high-risk PE treated with the EkoSonic Endovascular System. Operative bleeding risk, different management modalities, and post-PE-therapy presumptive complications were assessed before PE treatment. **Results:** Procedural success was achieved in 100% of cases. We observed a marked improvement in clinical and PE hemodynamics. One major bleeding, defined as life-threatening, required surgical intervention; four moderate bleedings, defined as bleeding without hemodynamic compromise, required intervention such as drainage. Minor bleeding was managed conservatively. **Conclusions:** Catheter-directed therapies may be an alternative to systemic reperfusion therapies for selected postoperative intermediate–high-risk PE-vulnerable populations.

## 1. Introduction

Pulmonary embolism (PE) is the third leading cardiovascular disease causing death worldwide [1]. It has heterogeneous triggers, and its management remains challenging. Challenges include the increasing aging population; comorbidities, particularly cancer and postoperative status; its various clinical presentations; early recognition and diagnosis; referral structures; processes; the complexity of the disease in acute treatment; navigating heterogeneous strategies for immediate and long-term anticoagulation, as well as advanced therapies between centers; subspecialty; guidelines differing from country to country; and long-term outcomes [2].

The current guidelines provide differing recommendations, and many clinical problems remain unresolved, such as the management of postoperative patients with intermediate–high-risk PE [1,3,4].

After diagnosis, risk stratification guides treatment [1,5], and systemic thrombolysis (ST) is an effective and widely used therapy for patients with high-risk and selected intermediate–high-risk PE because it rapidly improves pulmonary perfusion and hemodynamics.

However, the use of thrombolytic therapy is limited by a substantial risk of major bleeding and intracranial hemorrhage. This risk is particularly relevant in patients with recent surgery, in whom thrombolysis is often contraindicated or avoided due to postoperative bleeding concerns. Current guidelines still recommend ST as the first line of therapy in intermediate–high-risk PE [1]. This recommendation is based on the current evidence that fibrinolytic therapy decreases the hemodynamic decompensation rate but increases the risk of major hemorrhage and stroke. In the PEITHO study, extracranial bleeding occurred in 32 patients (6.3%) in the tenecteplase group compared to six patients (1.2%) in the placebo group (*p* > 0.001), and hemorrhagic stroke occurred in 2.4% and 0.2% of patients, respectively [6]. In addition, the decrease in overall mortality was not significant in hemodynamically stable patients with acute PE, and an increased risk of major hemorrhage and fatal or intracranial bleeding was observed in the overall population [7]. Moreover, patients with preoperative frailty have an additional higher risk of 1-year mortality in the early postoperative period [8].

Further, it has been estimated that more than half of patients with high-risk PE do not receive ST because of a perceived increased risk of bleeding [7,9]. Anticoagulation (AC), surgical embolectomy, or catheter-directed therapy (CDT) should be considered in such cases [9,10].

Postoperative patients represent a population with an increased risk of venous thromboembolism and an elevated risk of bleeding. Therefore, the management of PE in this group is challenging, and clinicians often face the dilemma of balancing the benefits of reperfusion therapy with the potential for serious bleeding complications. The high incidence (0.3–30%) and mortality rate (16.9–31%) of PE in patients undergoing major surgical procedures is suggested by the findings of contemporary observational studies [11]. In addition, a meta-analysis of data on the epidemiology, management strategies, and guideline recommendations of postoperative PE is lacking. This gap in evidence in this subgroup creates uncertainty among clinicians, and routine practice often defaults to AC alone.

Several cohort studies have suggested that patients with intermediate–high-risk PE may benefit from CDT when ST is contraindicated [12,13,14,15,16,17,18]. CDT uses thrombus aspiration or lower doses of thrombolytics, delivering treatment locally, and has emerged in the last decade as a potential alternative to ST. It may offer a more favorable efficacy and safety profile in postoperative patients with a higher bleeding risk [15,19,20].

Nevertheless, evidence regarding the safety and efficacy of CDT, including EkoSonic Endovascular System (EKOS) therapy (Boston Scientific, Marlborough, MA, USA), in postoperative patients with PE remains limited, and data specifically addressing this vulnerable population are scarce.

## 2. The Aim of This Study

This study was conducted to assess the efficacy and safety of the EkoSonic Endovascular System in postoperative patients with intermediate- and high-mortality-risk PE with additional bleeding risk.

Owing to its superior safety outcomes compared with ST and favorable hemodynamic results compared to AC alone, the use of the EkoSonic Endovascular System is suggested to be a reasonable treatment modality in this vulnerable population. Patients with a very high bleeding risk were excluded and treated with AC alone.

## 3. Methods

We started a register study with a prospective design at Petrus Hospital, Wuppertal, Germany, and Schoen Clinic Neustadt in Holstein, Germany, between October 2020 and November 2025. Then, we conducted the current study with a retrospective design and included all patients with postoperative intermediate–high-risk and high-risk PEs who received USAT with EKOS TM (Boston Scientific, Marlborough, MA, USA). A standardized protocol was used across both centers, and the pulmonary embolism response leader concept was implemented. We obtained written informed consent from all patients who participated in this study, which was approved by the institutional review board at Luebeck University (reference number 21–172). It is important to note that this study had a retrospective design. However, a primary endpoint was pre-specified for the ongoing prospective study.

Postoperative patients who suffered from acute dyspnea were screened for PE using echocardiography. After confirmation of PE with pulmonary contrast-enhanced computed tomography (CT), further risk stratification according to the Guidelines of the European Society of Cardiology (ESC) was performed. Patients with RV-strain, elevated N-terminal pro-brain natriuretic peptide (NTproBNP) and/or Troponin, and a simplified pulmonary severity index score (sPESI) of at least 1 point were stratified into an intermediate–high-risk PE group, whereas those with prolonged hypotension, shock, or cardiopulmonary resuscitation were stratified into a high-risk PE group. All patients suffered from a large thrombus burden within the main stem, right, and left pulmonary artery (central PE). In addition, right heart strain was observed in the CT cross-section for all patients. Visualized interpretation without software analysis of the findings was performed.

## 4. Study Population

- **Inclusion criteria**

Patients were eligible to participate if they had postoperative PE (CT-confirmed PE, post-surgical or interventional procedure, and mostly triggered by the procedure, such as immobility, dehydration, etc.), and a surgical region suitable for compression or drainage if bleeding occurred.

PE risk stratification:

- High-risk PE (prolonged systemic arterial hypotension, cardiogenic shock, or resuscitation).
- Intermediate–high-risk PE (Simplified Pulmonary Embolism Severity Index (sPESI) ≥ 1; RV strain with RV/LV ratio of 0.9 mm; and positive cardiac markers).

- **Exclusion criteria**

The exclusion criteria were as follows:

- Active intracranial or intraspinal bleeding within 6 months.
- Ischemic stroke within 3 months.
- Major and spinal cord surgery within 7 days.
- Recent active bleeding from a major organ.
- Platelets < 50,000/mL.

- **Intervention description**

The EKOS TM system was implanted via fluoroscopic guidance in the catheterization laboratory by interventional cardiologists with continuous hemodynamics and ECG monitoring.

During the procedure, AC was initiated with full-dose intravenous unfractionated heparin with a target-activated PTT of 60–80 s. In addition, all patients received 6 mg of tissue plasminogen activator (rtPA) over 6 h at a rate of 1 mg/h per catheter, and after 6 h of rtPA infusion and EKOS ultrasound, the therapy was stopped. The catheter was removed 6 h from the start of therapy, and the puncture site was compressed using a femoral compression system 4 h after therapy.

- **Post-intervention management**

Post-USAT patients received unfractionated heparin or were directly switched to vitamin K antagonists or direct anticoagulants according to bleeding risk. Finally, the patients were transferred to a normal ward.

**Figure 1:**
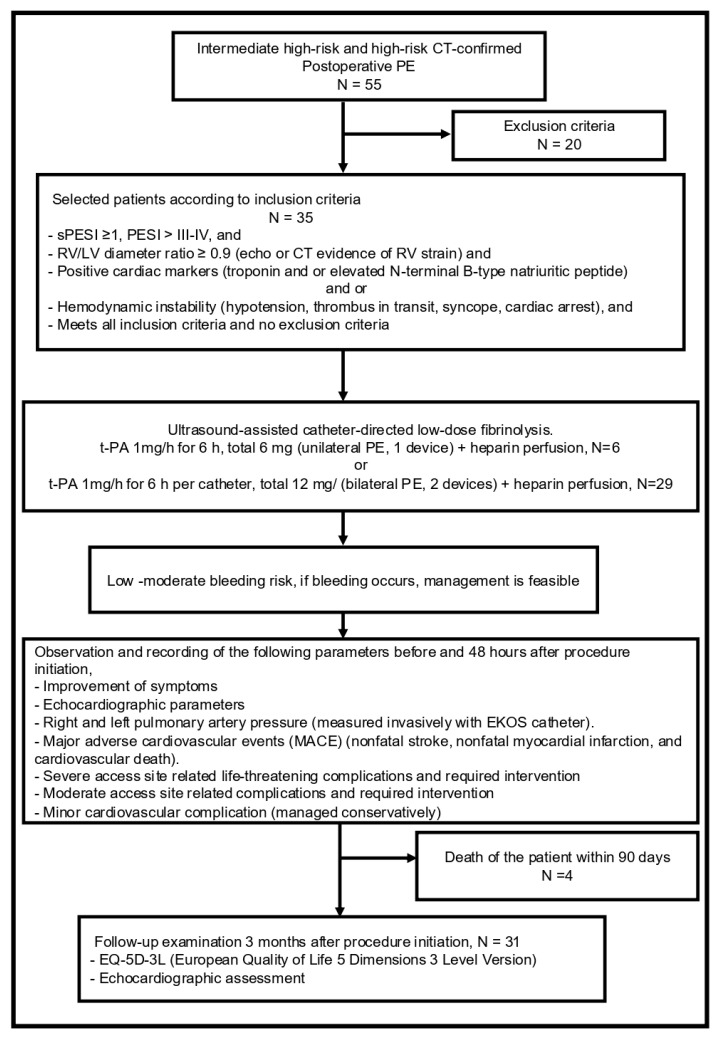
Study flow chart. One hundred ten patients with acute, intermediate, high-, and high-risk pulmonary embolism (PE) were screened for eligibility, and fifty-five patients were eligible for enrolment. CT: computed tomography; EKOS: EkoSonic endovascular system; PE: pulmonary embolism; RV/LV: right ventricle/left ventricle; sPESI: simplified pulmonary embolism severity index; EQ-5D-3L: EuroQol 5-Dimensional 3-Level; t-PA: tissue-type plasminogen activator.

Invasive pulmonary artery pressure was measured directly before (baseline) and after therapy (6 h). Using transthoracic echocardiography (TTE), RV-function, sPAP, RV/LV ratio, and NTproBNP or Troponin I were documented at baseline and 24 h after therapy (Figure 1).

All patients received unfractionated heparin with a target partial thromboplastin time (PTT) of 60–80 s. If the patients hemodynamically deteriorated despite AC, USAT was performed. Patients with a high bleeding risk were excluded and managed with AC alone.

The post-procedural anticoagulation strategy was based on the type of operation, bleeding risk, and clinical judgment. Patients with mild bleeding risk were treated with direct oral anticoagulants (DOACs). Patients with moderate or high bleeding risk were treated first with unfractionated heparin (UFH) or low-molecular-weight heparin (LMWH), followed by direct oral anticoagulants.

**Primary efficacy outcome**

Primary efficacy outcomes were the early mortality rate and the early venous thromboembolism (VTE) recurrence rate.

**Primary safety outcome**

**Figure 2:**
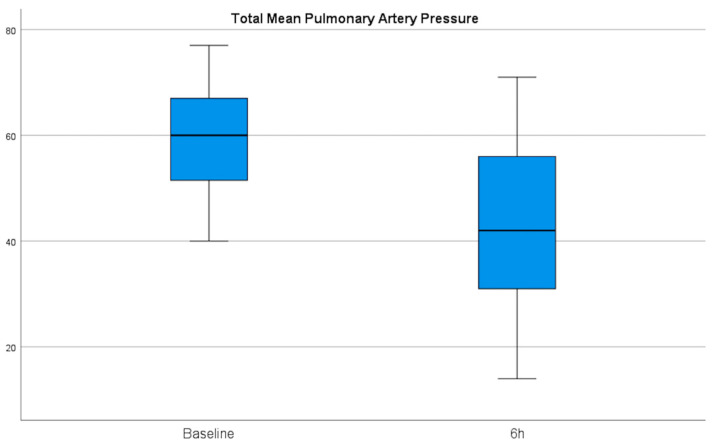
Invasive measurement of pulmonary artery pressure from baseline to 6 h after therapy showed a significant reduction. A decrease in the mean pulmonary artery pressures from 29 mmHg at baseline to 20 mmHg, with a mean difference of 9 mmHg, was observed.

Primary safety outcomes were major, moderate, and minor bleeding events. Major bleeding, defined as life-threatening bleeding, required surgical intervention and intensive care therapy; moderate bleeding, defined as bleeding without hemodynamic compromise, required intervention such as drainage; and minor bleeding, which was conservatively managed, and all-cause in-hospital mortality (Figure 2).

**Secondary outcomes**

For secondary outcomes, compared to baseline, indicators of USAT efficacy were a decrease in invasive sPAP, indirect measurements obtained via echocardiography, and improvements in RV function and the RV/LV ratio, measured by TTE 48 h after initiating therapy and 90 days after.

**Follow-up duration**

For follow-up examination 3 months after procedure initiation, we performed an echocardiography and quality of life assessment using

EQ-5D-3L questionary (European Quality of Life 5 Dimensions 3 Level Version).

### Statistical Analysis

Values are presented as numbers and proportions for qualitative variables or means and standard deviations, and as median (minimum–maximum) values for quantitative variables. Quantitative variables were checked for normality using the Shapiro–Wilk test. As the data were not normally distributed, non-parametric tests were used. All tests were bilateral, and a *p*-value of 5% was the limit of statistical significance. Analysis was performed using the statistical package software IBM-SPSS version 27. Within-group comparison was evaluated using the Wilcoxon signed rank test for two-time point comparisons of pulmonary artery outcomes. For more than two-time point comparison, the Friedman test was used for echocardiography outcomes. In the case of a significant difference, pairwise comparison between the two-time points was conducted while adjusting the *p*-value for multiple comparisons using Bonferroni correction. Correlation between the quantitative variables was analyzed using the Spearman correlation coefficient.

## 5. Results

The flowchart included data on 55 postoperative patients with PE, with 35 patients included and 20 patients excluded; 31 patients were followed up, and 4 patients died within 90 days and were lost to follow-up.

The baseline characteristics of the population were a mean age of 66 years, and the mean body mass index (30 kg/m^2^) was consistent with an obese patient population. Common comorbidities for PE included hypertension (23%), anemia (51%), active cancer (22%), and chronic renal disease (22%) (Table 1).

All patients had symptomatic PE. The duration of PE symptoms was within 14 days. The diagnosis was confirmed using CT-Thorax with contrast. Intermediate- and high-risk PEs were observed in 29 (89%) and 4 (11%) patients, respectively (Table 2 and Table 3).

The right femoral venous access was most frequently used for device placement in 20 (57%), left femoral vein in 7 (20%), right jugular vein in 3 (8.5%), and right brachial vein in 5 (23%) of the patients. All devices were successfully placed (100%). Bilateral devices were placed in 26 (82%) of the patients. Bilateral catheters were placed through a double-access site in every patient to treat bilateral PE. The mean total dose of t-PA was 6 mg for unilateral PE or 12 mg for bilateral PE. Completed infusion of t-PA was achieved in 35 (100%) patients. The duration of investigation was 10.212 ± 7.4178/minute, and radiation dosage was 2092.688 ± 4106.5534 cGY/cm^2^ (centi-gray per square centimeter).

## 6. Discussion

Postoperative patients have a higher risk of PE, which remains an Achilles’ heel in PE management. They have an additional bleeding risk, acute setting, recent surgery, comorbidities such as anemia, dehydration, renal failure, cancer, and high BMI, longer intensive care stay, and concomitant high doses of anticoagulant treatment.

In this cohort of postoperative patients with intermediate–high-risk PE treated with EKOS therapy, outcomes were acceptable from a safety perspective: one major access-site bleed and four moderate post-intervention bleeds without residual complications. There were four deaths within 90 days, driven by advanced cancer and comorbidity, yielding an early mortality rate of 11%; no early venous thromboembolism (VTE) and no recurrence of PE were observed. Procedural success (EKOS) reached 100%. These findings align with the literature trend that CDT/USAT approaches can be feasible and reasonably safe in this high-risk group, even when bleeding risk is elevated. We assessed bleeding complications according to management as follows:

One patient with intermediate–high-risk PE experienced retroperitoneal bleeding after the initiation of therapy, which was managed with successful surgical intervention. Four patients with intermediate–high-risk PE died within 90 days due to comorbidities and active cancer. We infer the cause of death due to acute illness with advanced comorbidities. We observed that 23 (67%) patients suffered from anemia, two (7%) patients suffered from mucosal bleeding, two (7%) patients had hematoma and pseudoaneurysm related to the access site, one (4%) patient suffered from knee bleeding, and one from pneumothorax.

**Figure 3:**
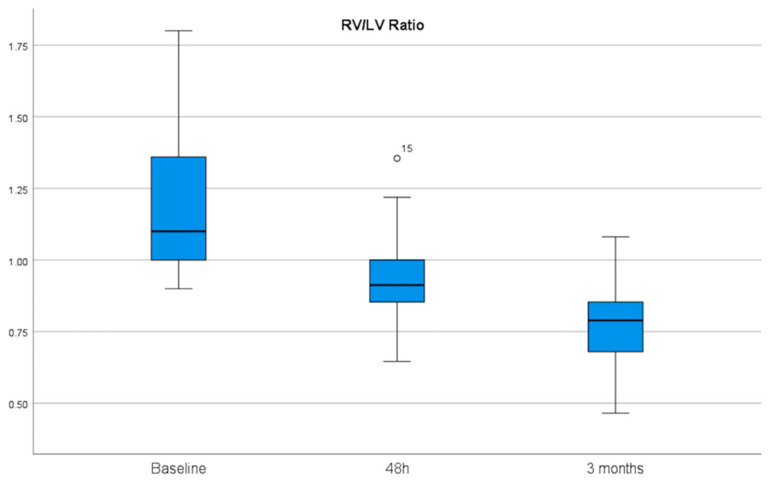
Echocardiographic RV/LV ratio measured from baseline to 48 h and 90 days after therapy initiation showed significant improvement. The mean RV/LV ratio decreased from 1.2 cm at baseline to 0.92 cm at 48 h and to 0.76 cm at the 90-day follow-up (*p* > 0.001). RV/LV: right ventricle/left ventricle.

**Figure 4:**
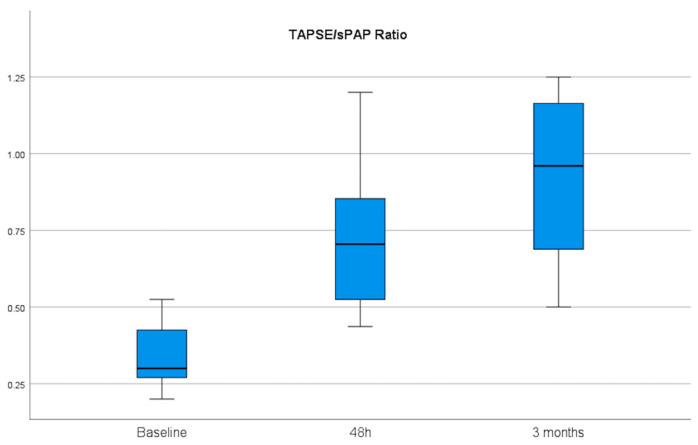
Echocardiographic TAPSE/sPAP ratio as an indicator for recovery of RV measured from baseline to 48 h and 90 days after therapy initiation showed significant improvement from 0.33 at baseline to 0.7 at 48 h and to 0.93 at 90 days, with *p*-value > 0.000. sPAP: systolic pulmonary artery pressure; TAPSE: tricuspid annular plane systolic excursion.

Regarding effectiveness, after 6 h of EKOS initiation, there was a significant reduction in the right systolic, diastolic, and mean pulmonary artery pressures from 46, 21, and 29 mmHg at baseline to 31, 15, and 20 mmHg, with mean differences of 15, 6, and 9, respectively. Additionally, we estimated a decrease in the left systolic, diastolic, and mean pulmonary artery pressures from 45, 19, and 29 mmHg at baseline to 31, 15, and 21 mmHg, with mean differences of 14, 4, and 8 mmHg, respectively (Table 4) (Figure 2). Echocardiographic indices showed a meaningful and sustained improvement in RV/LV ratio, which decreased from 1.2 cm at baseline to 0.92 cm at 48 h and to 0.76 cm at the 90-day follow-up (*p* > 0.001). The RV diameter decreased from 46 mm at baseline to 37 mm within 48 h, with a mean difference of 9, and to 33 at the 90-day follow-up, with a mean difference of 13. With TAPSE, a recovery of RV systolic function from 16 mm at baseline to 22 mm within 48 h and to 23 mm at the 90-day follow-up was observed (Table 5) (Figure 3 and Figure 4).

**Figure 5:**
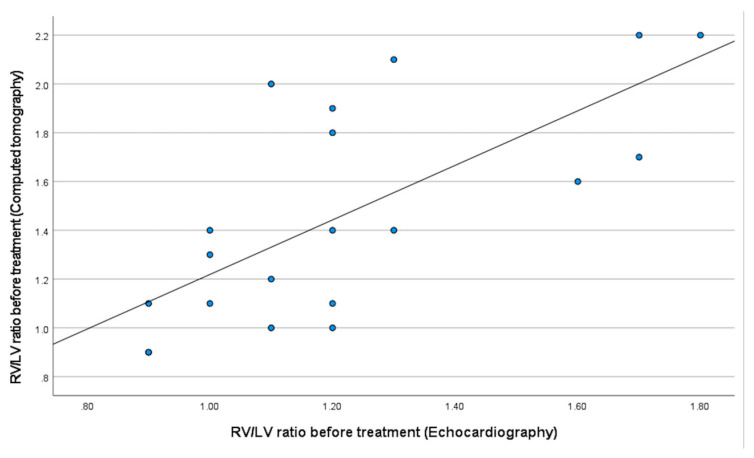
Weak correlation was observed between the RV/LV ratio measured in echocardiography and computer tomography before initiation of treatment. RV/LV: right ventricle/left ventricle.

A weak correlation was observed between RV/LV measurements by echocardiography and prior CT assessment before treatment, consistent with heterogeneity across imaging modalities (Figure 5). Interestingly, postoperative patients with PE treated with EKOS demonstrated longer ICU and hospital stay compared to other PE groups [15] Confirmation of PE with CT after the postoperative procedure was, on average, 5.7 days.

We observed an improvement in NT-Pro-BNP and lactate from 5385.91 ± 7316.39 pg/mL and 2.13 ± 1.53 mmol/L before therapy to 4578.81 ± 7396.56 pg/mL and 1.79 ± 2.39 mmol/L after EKOS therapy, respectively. On the other hand, hemoglobin decreased from 11.65 ± 2.86 g/dL before therapy to 10.19 ± 2.27 g/dL after EKOS therapy.

Procedure-related outcomes (*n* = 35), with length of stay of 13.21 ± 13.059/days, ICU (intermediate care unit) stay of 2.94 ± 7.175/days, complications rate of 9 (25%), and no serious or severe adverse events potentially related to device, were observed; one (2.8%) serious and severe adverse event potentially related to t-PA was observed, and four (11%) patients with moderate bleeding events, which required intervention, were observed. The mean improvement in symptoms was 80% on the 90-day follow-up questionnaire, with a mean quality of life of 95 using the EQ-5D-3L questionnaire (where 100 = the best health you can imagine, and 0 represents the worst health you can imagine).

This study did not apply established bleeding scores in isolation to guide anticoagulation decisions, consistent with guidelines (e.g., ESC) that warn against relying solely on bleeding scores, since many high-bleeding-risk factors coincide with high thrombotic risk [21].

It is important to take preventive measures in this high-risk group. Postoperative patients usually require prolonged monitoring, which may help in the early detection of PE. Saitoh et al. investigated PE incidence in 225 patients who underwent cardiovascular surgery. Although preventive measures such as postoperative antithrombotic therapy and postoperative intermittent pneumatic compression therapy were undertaken, the incidence of postoperatively developed DVT was 7.3% in this study [22].

Therefore, preventive strategies should be implemented through peri-interventional risk assessment, including clinical, ultrasound, and radiological preprocedural work-up, and a team approach to hindering PE. Van Lier et al.’s study concluded that patients who are overweight, have undergone surgery for malignancy, have a history of cerebrovascular disease and thromboembolic diseases, have intraoperative blood transfusions, and delayed use of thromboprophylaxis were at higher risk for postoperative pulmonary embolism. Delayed use of thromboprophylaxis was associated with a four-fold increase in risk (OR 4.1; 95% CI: 2.1–7.7) [23].

In addition, Huber et al. stated that the rate of postoperative PE increased by 30% when PEs that occurred within 30 days of hospital discharge were considered. This provides a useful basis for prolonged prophylactic measures after a hospital stay [24]. Shaw et al. suggested a decision algorithm for periprocedural anticoagulation management. Accordingly, direct oral anticoagulants can be interrupted 1 day before low-bleed-risk procedures and 2 days before high-bleed-risk procedures. Longer interruptions may be needed for patients with renal dysfunction on dabigatran. Anticoagulants can be resumed 24 h after low-bleed-risk procedures and 48–72 h after high-bleed-risk procedures [25].

However, if PE occurs, the decisions regarding periprocedural AC management must balance thromboembolic and procedural higher bleeding risks. In addition, anticoagulant pharmacokinetics, comorbidities, and patient characteristics should be considered. The post-procedural anticoagulation strategy was based on the type of operation, bleeding risk, and clinical judgment. Patients with mild bleeding risk were treated with direct oral anticoagulants. Patients with a moderate or high bleeding risk were treated first with UFH or LMWH and then with direct oral anticoagulants. The use of UFH or DOACs did not influence bleeding event rates in this study. One patient suffered from hemothorax under UFH, whereas other patients were under DOACs.

According to the ESC guideline, a recent major surgery is a relative contraindication to ST. In postoperative patients with intermediate–high-risk PE, small doses of local lytics or mechanical percutaneous embolectomy may be a safer option. However, even with the anticipation of a high risk of bleeding, ST with rtPA can be used as a life-saving treatment in the case of PE and in the absence of alternative management strategies [26,27].

USAT, a CDT established over the last decade, aims to reduce bleeding risk using a combination of ultrasound energy and low-dose thrombolysis.

Data concerning USAT are promising [12], even in frail elderly patients [28]. Additionally, improvement in right ventricular (RV) function and decreases in sPAP and the RV/LV ratio have been reported [15].

The FLASH registry results on the use of the FlowTriever (Inari Medical) thrombus aspiration system reported a major bleeding rate of 1.4% within 48 h in a population of patients with PE and a mean age of 61 years [29].

In the EXTRACT-PE trial, among patients with a mean age of 59.8 years, the rate of major bleeding within 48 h using the Indigo mechanical thrombectomy system (Penumbra, Inc. (Alameda, CA, USA)) was also 1.7% [14].

The Peerless trial compared large-bore mechanical thrombectomy (LBMT) (INARI) with CDT. It demonstrated no significant differences in mortality, intracranial hemorrhage, or major bleeding between the two strategies [13].

Farmakis et al. analyzed the US registry data of over 980,000 patients with PE aged ≥65 years (28.0% were frail). Interestingly, among frail high-risk patients, CDT, compared with ST, was associated with reduced major bleeding (odds ratio [OR] 0.51, 95% confidence interval [CI]: 0.41–0.63), lower intracranial hemorrhage (1% vs. 3%), and in-hospital mortality rate (OR 0.36; 95% CI:0.28–0.46) [30].

Al-Khadra et al. analyzed a database of in-hospital outcomes of patients with PE who underwent mechanical thrombectomy (MT) compared to those who underwent ST. Patients with PE who underwent MT had significantly lower mortality compared to those who were treated using ST [31]. De Gregorio et al. performed MT, followed by CDT, in patients with acute unstable PE with promising results [32].

Loyalka et al. performed a meta-analysis of 75 studies (41 of CDT; 34 of surgical pulmonary embolectomy (SE)). In 1650 patients undergoing CDT and 1101 undergoing SE in more critically ill populations, both CDT and SE were associated with satisfactory outcomes. SE was associated with greater absolute postprocedure mortality than CDT [33]. Percy et al. analyzed 58,974 registry patients undergoing ST, CDT, and SE for acute PE from 2010 to 2014. The rates of major bleeding and intracranial hemorrhage were the highest in the ST group, whereas mortality occurred in 19.8% of patients undergoing SE [34].

Su et al. performed another meta-analysis on the clinical outcomes of patients with PE who received anticoagulation (AC) compared to ST. The addition of CDT to AC increases the risk of major bleeding but improves mortality outcomes for submassive PE, whereas ST reduces recurrence and deterioration in acute intermediate PE with increasing minor bleeding [35].

However, complications during LBMT include hemodynamic decompensation, respiratory failure, alveolar hemorrhage, pulmonary artery perforation, contrast-induced acute kidney injury, and hematomas at the vascular access site.

Access-related complications are the most frequent and have a direct impact on outcome and mortality, potentially leading to prolonged in-hospital stays, increased blood transfusions, and in-hospital mortality. Preventive and risk factor modification strategies are needed to improve safety outcomes and reduce complication burdens, such as the use of low-profile sheaths, US-guided puncture, and advances in vascular closure techniques. However, if a complication occurs, a streamlined pathway and algorithms should be implemented to standardize the management strategy.

CDT requires specialized interventional expertise, adequate infrastructure, and dedicated monitoring during and after the procedure. However, the overall results suggest that CDT is a safer option for postoperative patients with intermediate–high-risk PE, even during on- and off-hours [36].

The obtained results confirm those stated in the literature regarding CDT trials, showing fewer bleeding events in the postoperative vulnerable group. However, underlying comorbidities could explain the longer hospital stay.

This study highlights the role of CDT in this particular PE-vulnerable population, and more data concerning the use of CDT in postoperative PEs are required.

**Figure 6:**
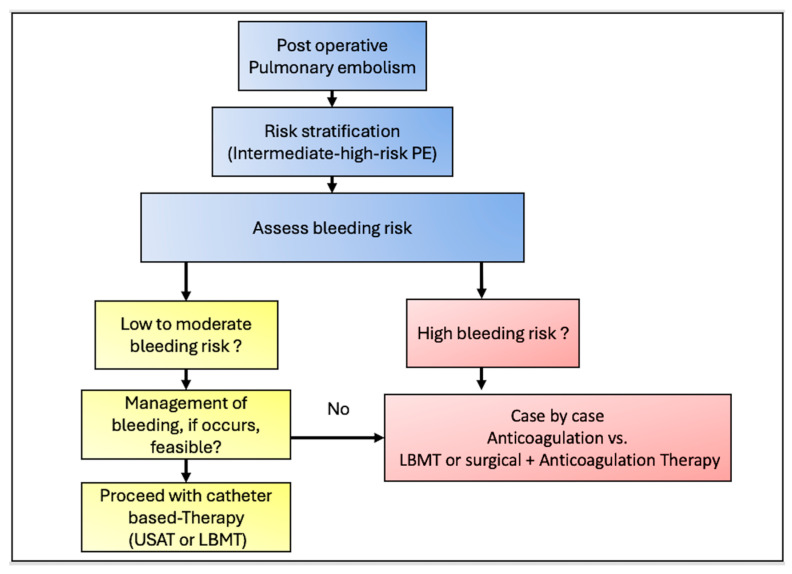
Step-by-step approach to intermediate–high-risk postoperative patients’ management. It is important to note that this algorithm is derived from the daily clinical practice. Future studies to assess the validity are necessary, and to further improve management algorithms in this particular vulnerable PE group. LBMT: large-bore mechanical thrombectomy; PE: pulmonary embolism; Ultrasound-accelerated thrombolysis (USAT).

Finally, an individualized management strategy in vulnerable patients with PE that considers the risk/benefit ratio of the patient’s comorbidities, clinical characteristics, hemodynamics, and bleeding risk is the key to improving outcomes. Cohort studies or meta-analyses comparing different management strategies in this vulnerable group are scarce. Based on our findings, we propose a management algorithm tailored specifically to this high-risk population (Figure 6). Patients are eligible to participate if they have postoperative PE (CT-confirmed PE, post-surgical or interventional procedure, and mostly triggered by the procedure, such as immobility, dehydration, etc.). The second step is to perform the risk stratification. All patients with high-risk or intermediate–high-risk should be further stratified to assess the bleeding risk and surgical procedure. Patients with very high bleeding risk and or a non-compressible surgical region, such as a spinal cord procedure, should be managed with AC alone or may benefit from LBMT, while patients with a surgical region suitable for compression or drainage if bleeding occurs, such as Knee bleeding, and with low to moderate bleeding risk may benefit from EKOS or LBMT. It is important to note that this algorithm is derived from daily clinical practice. Future studies to assess the validity are necessary to further improve management algorithms in this vulnerable PE group.

In addition, ongoing clinical trials may influence future guideline recommendations, particularly in determining whether CDT confers a clear clinical benefit in postoperative patients with intermediate–high-risk PE.

The main limitations of this study are its two-centered, non-randomized, retrospective design and relatively small sample size for the USAT investigation; therefore, the results should be interpreted carefully. In addition, we used only EKOS therapy in this study. Using other CDTs, such as LBMT devices, could help in understanding the proper indication for each device in daily clinical practice.

Moreover, this study has statistical analysis limitations of only one group, which resulted in complete separation of the outcomes and prevented the use of conventional logistic regression to adjust for potential confounders such as anemia frequency, renal function, and PE risk. Although this limits the ability to perform adjusted modeling, the one group reflects the observed data.

There is a need for multicenter, randomized studies with larger samples to compare EKOS/USAT against other CDT and mechanical thrombectomy strategies, specifically in postoperative patients, and to allow more robust multivariable analyses. Further work should define patient selection criteria, bleeding risk stratification, and standardized management algorithms to refine who benefits most from CDT versus other modalities.

## 7. Conclusions

Catheter-directed therapy using EKOS may be a safer alternative to systemic reperfusion therapies for postoperative intermediate–high-risk PE-vulnerable populations with low to moderate bleeding risk.

An individualized management strategy in vulnerable patients with PE that considers the risk/benefit ratio with the patient’s comorbidities, clinical characteristics, hemodynamics, and bleeding risk is the key to improving outcomes.

**The main perspectives of this study are as follows:**

1. Catheter-directed therapies, such as small doses of local lytics or mechanical percutaneous embolectomy, may be a safer alternative to systemic reperfusion therapies for postoperative intermediate–high-risk PE-vulnerable populations.
2. Ultrasound-accelerated endovascular thrombolysis can be conducted in this vulnerable group with low to moderate bleeding risk.
3. This study initiates a discussion in the literature as to which CDTs are suitable for certain postoperative patients to improve outcomes.

## Figures and Tables

**Table 1:** Baseline Demographics and Comorbid Conditions Characteristics (*n* = 35).

Age/year	66.4377 ± 12.39
Body mass index kg/m^2^	30.711 ± 7.33
Female	17 (49%)
Ethnicity/race	White
Comorbid diseases such as heart failure	26 (74%)
Hypertension defined as blood pressure > 140/90 mmHg	8 (23%)
Diabetes Mellitus	0
Smoking	2 (5.7%)
Immobility	13 (37%)
Previous DVT	3 (8.6%)
Previous PE	3 (8.6%)
Atherosclerotic cardiovascular disease	3 (8.6%)
Anemia with hemoglobin < 13 mg/dL in male and <12 mg/dL in female	18 (51%)
Stroke	2 (5.7%)
Active cancer	8 (22%)
Chronic pulmonary disease	5 (14%)
Chronic kidney disease (GFR < 90 mL/min)	8 (22%)
Hyperlipidemia defined as (cholesterol > 200 mg/dL, LDL > 116 mg/dL)	3 (8.6%)
Congenital Blood disease such as thalassemia, hemophilia, and or sickle-cell disease.	1 (3%)

DVT: deep vein thrombosis; GFR: glomerular filtration rate; Kg: kilogram; PE: pulmonary embolism.

**Table 2:** Characteristics of PE (*n* = 35).

Dyspnea III (symptoms with minimal exertion)	22 (61%)
Dyspnea IV (symptoms at rest)	10 (27%)
Cardiopulmonary resuscitation	2 (5.6%)
Syncope	1 (2.8%)
High risk	4 (11%)
Intermediate–high risk	31 (89%)
**Diagnosed DVT**	
Right	6 (17%)
Left	2 (5.7%)
**Oxygen saturation/%**	87.70%
**Breathing frequency/min**	22.27 ± 4.1
**Heart rate/min before EKOS therapy**	100.86 ± 16.59
Systole blood pressure/mmHg	133.08 ± 28.37
Diastole blood pressure/mmHg	78.24 ± 18.41
Right PE	4 (11%)
Left PE	1 (2.8%)
Bilateral PE	29 (89%)
**Electrocardiogram**	
Sinus rhythm	6 (17%)
Sinus tachycardia (Heart rate > 90/min)	23 (66%)
Atrial flatter	0
Atrial fibrillation	5 (14%)
**Troponin: ng/mL**	721.09 ± 845.62
**NT-Pro-BNP: pg/mL**	5385.91 ± 7316.39
**Lactate: >2.0 mmol/L**	2.13 ± 1.53
**Hemoglobin: g/dL**	11.65 ± 2.86
**CRP: mg/dL**	61.78 ± 42.79
**Creatinine: mL/dL**	0.93 ± 0.23
**GFR: mL/min**	76.46 ± 27.41
**TSH: mIU/L**	1.82 ± 1.02

DVT: deep vein thrombosis; CRP: C-reactive Protein; GFR: glomerular filtration rate; TSH: thyroid stimulating hormone.

**Table 3:** Disease, type of operation, and postoperative PE diagnosis/day. (*n* = 35).

Type of Operation	Number of Patients	Postoperative Diagnosis of PE/Days
Open surgical colon resection	5 (11%)	11, 2, 4, 14, 30
Mechanical carotid thrombectomy	1 (2.8%)	8
Cystoscopy	1 (2.8%)	2
Quadriceps tendon repair	1 (2.8%)	6
STEMI and stenting of left anterior descending artery	1 (2.8%)	7
Right ankle joint replacement	1 (2.8%)	3
Herniotomy	1 (2.8%)	2
Hemothorax, VATS, left re-thoracoscopy and evacuation	1 (2.8%)	2
Hip joint replacement	4 (11%)	1, 1, 1, 2, 2
Elbow joint repair and fixation	1 (2.8%)	4
Distal radius fixation	1 (2.8%)	2
Knee joint replacement	4 (11%)	4, 3, 3, 13
Cholecystectomy	5 (14%)	2, 2, 2, 3, 2
Shoulder prothesis replacement	2 (5.7%)	2,1
Whipple operation	3 (8.6%)	2, 3, 2
Varicose ligation	1 (2.8%)	2
Uterine prolapse with hydronephrosis, treated with a pessary	1 (2.8%)	29

STEMI: ST-elevation myocardial infarction; VATS: Video-assisted thoracoscopic surgery.

**Table 4:** Primary pulmonary artery pressure efficacy outcomes.

PA/mmHg	Baseline	6 h	*p*-Value
Right systolic pulmonary artery pressure	46.91 ± 10.104	31.50 ± 11.741	0.000
Right diastolic pulmonary artery pressure	21.65 ± 11.052	15.15 ± 7.103	0.022
Right mean pulmonary artery pressure	29.22 ± 6.317	20.80 ± 7.654	0.001
Left systolic pulmonary artery pressure	45.38 ± 8.992	31.27 ± 11.949	0.002
Left diastolic pulmonary artery pressure	19.29 ± 6.051	15.73 ± 8.371	0.155
Left mean pulmonary artery pressure	29.52 ± 4.781	21.33 ± 9.715	0.011
Total mean systolic pulmonary artery pressure	59.76 ± 9.767	42.80 ± 17.301	0.006

PA/mmHg: Pulmonary artery/millimeters of mercury.

**Table 5:** Echocardiography efficacy outcomes.

Echocardiography	Baseline	48 h	90 Days	*p*-Value
RVEDD, mm	46.05 ± 6.074	37.45 ± 4.957	33.50 ± 3.940	0.000
LVEDD, mm	37.26 ± 6.715	40.63 ± 7.025	44.37 ± 6.914	0.000
RV/LV ratio	1.2156 ± 0.29312	0.9290 ± 0.17153	0.7605 ± 0.14816	0.000
TAPSE, mm	16.32 ± 5.657	22.11 ± 3.017	23.47 ± 3.062	0.001
sPAP, mmHg	49.57 ± 7.965	33.26 ± 8.619	25.65 ± 6.336	0.000
TAPSE/sPAP	0.3316 ± 0.10175	0.7021 ± 0.21039	0.9312 ± 0.24673	0.000

LVEDD: left ventricular end-diastolic diameter; MM: millimeter, RVEDD: right ventricular end-diastolic diameter; RV/LV: right ventricle/left ventricle; sPAP: systolic pulmonary artery pressure; TAPSE: tricuspid annular plane systolic excursion.

## Data Availability

All data related to this article are available on request. The paper is not under consideration elsewhere; none of the paper’s contents have been previously published; all authors have read and approved the manuscript.

## Abbreviations

Blood pressure	BP
Body-mass-index	BMI
Catheter-directed-thrombolysis	CDT
Contrast-enhanced computer tomography	CT
Direct oral anticoagulants	DOACs
European Society of Cardiology	ESC
Heart rate	HR
Hemoglobin	Hb
Large-bore mechanical thrombectomy	LBMT
Low molecular weight heparin	LMWH
Left ventricle end-diastolic diameter	LVEDD
N-terminales pro brain natriuretic peptide	NTproBNP
Partial thromboplastin time	PTT
Pulmonary embolism	PE
Recombinant tissue plasminogen activator	rt-PA
Respiratory rate	RR
Right ventricle	RV
Right ventricle end-diastolic diameter	RVEDD
simplified pulmonary severity index score	sPESI
Standard deviation	SD
Systolic pulmonary artery pressure	sPAP
Transthoracic echocardiography	TTE
Tricuspid annular plane systolic excursion	TAPSE
Unfractionated heparin	UFH
Ultrasound-accelerated-endovascular thrombolysis	USAT

## References

[1] Konstantinides S.V., Meyer G., Becattini C., Bueno H., Geersing G.J., Harjola V.-P., Huisman M.V., Humbert M., Jennings C.S., Jiménez D. (2020). 2019 ESC Guidelines for the diagnosis and management of acute pulmonary embolism developed in collaboration with the European Respiratory Society (ERS): The Task Force for the diagnosis and management of acute pul-monary embolism of the. Eur. Heart J..

[2] Saha U., Arko S.B., Shama S.S., Gonzalez C. (2024). Pulmonary Embolism: Hidden in the Disguise of Atrial Flutter/Atrial Tachycardia. Cureus.

[3] Douillet D., Chouihed T., Bertoletti L., Roy P.-M. (2023). Pulmonary Embolism and Respiratory Deterioration in Chronic Cardiopulmonary Disease: A Narrative Review. Diagnostics.

[4] Giri J., Sista A.K., Weinberg I., Kearon C., Kumbhani D.J., Desai N.D., Piazza G., Gladwin M.T., Chatterjee S., Kobayashi T. (2019). Interventional Therapies for Acute Pulmonary Embolism: Current Status and Principles for the Development of Novel Evidence: A Scientific Statement From the American Heart Association. Circulation.

[5] de Wit K., D’aRsigny C.L. (2023). Risk stratification of acute pulmonary embolism. J. Thromb. Haemost..

[6] Meyer G., Vicaut E., Danays T., Agnelli G., Becattini C., Beyer-Westendorf J., Bluhmki E., Bouvaist H., Brenner B., Couturaud F. (2014). Fibrinolysis for patients with intermediate-risk pulmonary embolism. N. Engl. J. Med..

[7] Galié N., Manes A., Dardi F., Palazzini M. (2020). Thrombolysis in high-risk patients with acute pulmonary embolism: Underuse of a life-saving treatment in the real-world setting. Eur. Heart J..

[8] McIsaac D.I., Bryson G.L., van Walraven C. (2016). Association of Frailty and 1-Year Postoperative Mortality Following Major Elective Noncardiac Surgery: A Population-Based Cohort Study. JAMA Surg..

[9] Keller K., Hobohm L., Ebner M., Kresoja K.-P., Münzel T., Konstantinides S.V., Lankeit M. (2020). Trends in thrombolytic treatment and outcomes of acute pulmonary embolism in Germany. Eur. Heart J..

[10] Al-Terki H., Elhakim A., Mügge A. (2023). EKOS^TM^ in Octogenarians: The Safety and Efficacy of Ultrasound-Accelerated Catheter-Directed Thrombolysis in Elderly Patients with Intermediate-High-Risk Pulmonary Embolism. J. Clin. Med..

[11] Danwang C., Temgoua M.N., Agbor V.N., Tankeu A.T., Noubiap J.J. (2017). Epidemiology of venous thromboembolism in Africa: A systematic review and meta-analysis protocol. BMJ Open.

[12] Kucher N., Boekstegers P., Müller O.J., Kupatt C., Beyer-Westendorf J., Heitzer T., Tebbe U., Horstkotte J., Müller R., Blessing E. (2014). Randomized, Controlled Trial of Ultrasound-Assisted Catheter-Directed Thrombolysis for Acute Intermediate-Risk Pulmonary Embolism. Circulation.

[13] Jaber W.A., Gonsalves C.F., Stortecky S., Horr S., Pappas O., Gandhi R.T., Pereira K., Giri J., Khandhar S.J., Ammar K.A. (2025). Large-Bore Mechanical Thrombectomy Versus Catheter-Directed Thrombolysis in the Management of Intermediate-Risk Pulmonary Embolism: Primary Results of the PEERLESS Randomized Controlled Trial. Circulation.

[14] Sista A.K., Horowitz J.M., Tapson V.F., Rosenberg M., Elder M.D., Schiro B.J., Dohad S., Amoroso N.E., Dexter D.J., Loh C.T. (2021). Indigo Aspiration Sys-tem for Treatment of Pulmonary Embolism: Results of the EXTRACT-PE Trial. JACC Cardiovasc. Interv..

[15] Elhakim A., Knauth M., Elhakim M., Böhmer U., Patzelt J., Radke P. (2022). Using a Fibrinolysis Delivery Catheter in Pulmonary Embolism Treatment for Measurement of Pulmonary Artery Hemodynamics. Adv. Respir. Med..

[16] Lin D.S., Lin Y., Wu C., Lin H., Lee J. (2021). Midterm Prognosis of Patients With Pulmonary Embolism Receiving Catheter-Directed Thrombolysis or Systemic Thrombolysis: A Nationwide Population-Based Study. J. Am. Heart Assoc..

[17] Sadeghipour P., Jenab Y., Moosavi J., Hosseini K., Mohebbi B., Hosseinsabet A., Chatterjee S., Pouraliakbar H., Shirani S., Shishehbor M.H. (2022). Catheter-Directed Thrombolysis vs Anticoagulation in Patients With Acute Intermediate-High–risk Pulmonary Embolism: The CANARY Randomized Clinical Trial. JAMA Cardiol..

[18] Pruszczyk P., Klok F.K., Kucher N., Roik M., Meneveau N., Sharp A.S., Nielsen-Kudsk J.N.-K., Obradović S., Barco S., Giannini F. (2022). Percutaneous treatment options for acute pulmonary embolism: A clinical consensus statement by the ESC Working Group on Pulmonary Circulation and Right Ventricular Function and the European Association of Percutaneous Cardiovascular Interventions. EuroIntervention.

[19] Al-Terki H., Mügge A., Gotzmann M., Tiyerili V., Klein F., Franz M., Möbius-Winkler S., Elhakim A. (2023). The Safety and Efficacy of Ultrasound-Accelerated Catheter-Directed Thrombolysis in Patients with Intermediate–High-Risk Pulmonary Embolism: Bo-NE-Experience. J. Clin. Med..

[20] Al-Terki H., Lauder L., Mügge A., Götzinger F., Elhakim A., Mahfoud F. (2024). Ultrasound-assisted endovascular thrombolysis versus large-bore thrombectomy in acute intermediate-high risk pulmonary embolism: The propensity-matched EKNARI cohort study. Catheter. Cardiovasc. Interv..

[21] Van Gelder I.C., Rienstra M., Bunting K.V., Casado-Arroyo R., Caso V., Crijns H.J.G.M., De Potter T.J.R., Dwight J., Guasti L., Hanke T. (2024). 2024 ESC Guidelines for the management of atrial fi-brillation developed in collaboration with the European Association for Cardio-Thoracic Surgery (EACTS): Developed by the task force for the management of atrial fibrillation of the European Society of Cardi-ology (ESC), with the special contribution of the European Heart Rhythm Association (EHRA) of the ESC. Endorsed by the European Stroke Organisation (ESO). Eur. Heart J..

[22] Saitoh M., Kudo T., Watanabe T. (2024). Incidence of venous thromboembolism after cardiovascular surgery. Asian Cardiovasc. Thorac. Ann..

[23] van Lier F., van der Geest P.J., Hol J.W., Leebeek F.W., Hoeks S.E. (2012). Risk modification for postoperative pulmonary embolism: Timing of postoperative prophylaxis. Thromb. Res..

[24] Huber O., Bounameaux H., Borst F., Rohner A. (1992). Postoperative pulmonary embolism after hospital discharge: An underes-timated risk. Arch. Surg..

[25] Shaw J.R., Kaplovitch E., Douketis J. (2020). Periprocedural Management of Oral Anticoagulation. Med. Clin..

[26] Al-Ghamdi T.H., Jarrad A., Bashir A.Y., Lorf T., Obed A. (2020). Thrombolysis in Postoperative Pulmonary Embolism Following Liver Transplantation: A Case Report. Am. J. Case Rep..

[27] Yu Y., Zhai Z., Yang Y., Xie W., Wang C. (2017). Successful thrombolytic therapy of post-operative massive pulmonary embolism after ultralong cardiopulmonary resuscitation: A case report and review of literature. Clin. Respir. J..

[28] Zuin M., Bikdeli B., Ballard-Hernandez J., Barco S., Battinelli E.M., Giannakoulas G., Jimenez D., Klok F.A., Krishnathasan D., Lang I.M. (2024). International Clinical Practice Guideline Recommendations for Acute Pulmonary Embolism: Harmony, Dissonance, and Silence. J. Am. Coll. Cardiol..

[29] Toma C., Jaber W.A., Weinberg M.D., Bunte M.C., Khandhar S., Stegman B., Gondi S., Chambers J., Amin R., Leung D.A. (2023). Acute outcomes for the full US cohort of the FLASH mechanical thrombectomy registry in pulmonary embolism. EuroIntervention.

[30] Farmakis I.T., Barco S., Giannakoulas G., Keller K., Valerio L., Tichelbäcker T., Partovi S., Ahrens I., Konstantinides S.V., Hobohm L. (2023). A nationwide analysis of reperfusion therapies for pulmonary embolism in older patients with frailty. EuroIntervention.

[31] Al-Khadra Y., Missula V., Al-Bast B., Singanallur P., Al Tamimi R., Albast N., Abdu M., Deshpande R., Salih M., White P. (2024). Outcomes of Mechanical Thrombectomy Compared With Systemic Thrombolysis in Pulmonary Embolism: A Comprehensive Evaluation From the National Inpatient Sample Database. J. Endovasc. Ther..

[32] De Gregorio M.A., Guirola J.A., Kuo W.T., Serrano C., Urbano J., Figueredo A.L., Sierre S., Quezada C.A., Barbero E., Jiménez D. (2019). Catheter-directed aspiration thrombectomy and low-dose thrombolysis for patients with acute unstable pulmonary embolism: Prospective outcomes from a PE registry. Int. J. Cardiol..

[33] Loyalka P., Ansari M.Z., Cheema F.H., Miller C.C., Rajagopal S., Rajagopal K. (2018). Surgical pulmonary embolectomy and catheter-based therapies for acute pulmonary embolism: A contemporary systematic review. J. Thorac. Cardiovasc. Surg..

[34] Percy E.D., Shah R., Hirji S., Tartarini R.J., Yazdchi F., Harloff M., Kaneko T., Pelletier M.P. (2020). National Outcomes of Surgical Embolectomy for Acute Pulmonary Embolism. Ann. Thorac. Surg..

[35] Su Y., Zou D., Liu Y., Wen C., Zhang X. (2024). Anticoagulant Impact on Clinical Outcomes of Pulmonary Embolism Compared With Thrombolytic Therapy; Meta-Analysis. Clin. Cardiol..

[36] Schulten-Baumer J., Elhakim A., Radke P., Schuchert A., Stöcker B., Mezger M., Rawish E., Genske F., Stiermaier T., Eitel I. (2025). Safety and efficacy of thrombolysis with the EkoSonic Endovascular System for intermediate-high risk pulmonary embolism during on- and off-hours: A multicenter study. Clin. Res. Cardiol..

